# Combined effects of exercise training and *D*‐allulose intake on endurance capacity in mice

**DOI:** 10.14814/phy2.15297

**Published:** 2022-05-11

**Authors:** Takamasa Tsuzuki, Ryo Suzuki, Risa Kajun, Takako Yamada, Tetsuo Iida, Bingyang Liu, Teruhiko Koike, Yukiyasu Toyoda, Takayuki Negishi, Kazunori Yukawa

**Affiliations:** ^1^ 12942 Faculty of Pharmacy Meijo University Nagoya Aichi Japan; ^2^ Research and Development Matsutani Chemical Industry Co., Ltd Itami Hyogo Japan; ^3^ 12965 Department of Sports Medicine Graduate School of Medicine Nagoya University Nagoya Aichi Japan; ^4^ 12965 Research Center of Health, Physical Fitness and Sports Nagoya University Nagoya Aichi Japan

**Keywords:** endurance capacity, exercise training, glycogen, rare sugar

## Abstract

This study investigated the combined effects of exercise training and *D*‐allulose intake on endurance capacity in mice. Male C57BL/6J mice were fed either a control diet (Con) or a 3% *D*‐allulose diet (Allu) and further divided into the sedentary (Sed) or exercise training (Ex) groups (Con‐Sed, Con‐Ex, Allu‐Sed, Allu‐Ex, respectively; *n* = 6–7/group). The mice in the Ex groups were trained on a motor‐driven treadmill 5 days/week for 4 weeks (15–18 m/min, 60 min). After the exercise training period, all mice underwent an exhaustive running test to assess their endurance capacity. At 48 h after the running test, the mice in the Ex groups were subjected to run at 18 m/min for 60 min again. Then the gastrocnemius muscle and liver were sampled immediately after the exercise bout. The running time until exhaustion tended to be higher in the Allu‐Ex than in the Con‐Ex group (*p* = 0.08). The muscle glycogen content was significantly lower in the Con‐Ex than in the Con‐Sed group and was significantly higher in the Allu‐Ex than in the Con‐Ex group (*p* < 0.05). Moreover, exercise training increased the phosphorylation levels of adenosine monophosphate‐activated protein kinase (AMPK) in the muscle and liver. The phosphorylation levels of acetyl coenzyme A carboxylase (ACC), a downstream of AMPK, in the muscle and liver were significantly higher in the Allu‐Ex than in the Con‐Sed group (*p* < 0.05), suggesting that the combination of exercise training and *D*‐allulose might have activated the AMPK‐ACC signaling pathway, which is associated with fatty acid oxidation in the muscle and liver. Taken together, our data suggested the combination of exercise training and *D*‐allulose intake as an effective strategy to upregulate endurance capacity in mice. This may be associated with sparing glycogen content and enhancing activation of AMPK‐ACC signaling in the skeletal muscle.

## Graphical abstract

This study investigated the combined effects of exercise training and D‐allulose intake on endurance capacity in mice. Our data suggest the combination of exercise training and D‐allulose intake as an effective strategy to upregulate endurance capacity by enhancing lipid metabolism and sparing glycogen content in the skeletal muscle.

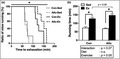

## 1 INTRODUCTION

Endurance capacity is important for performance in endurance athletes and for maintaining the quality of life in ordinary people. A determinant of endurance capacity is energy metabolism during exercise. The main fuels during exercise are carbohydrates and lipids (Brooks & Mercier, 1994). When exercise intensity is relatively low, fat is utilized as the main fuel for adenosine triphosphate (ATP) synthesis. The demand for carbohydrate utilization gradually increases with increase in the exercise intensity (Spriet, 2014). Glycogen depletion in the muscle results in exercise‐induced exhaustion due to limited glycogen stores in the body (Green, 1991; Karlsson & Saltin, 1971). The level of and ability to increase glycogen stores are crucial limiting factors for aerobic endurance. Moreover, increased fat oxidation leads to the sparing of glycogen consumption in the muscle and liver during exercise (Rennie et al., 1976). Thus, regulation of energy metabolism by increasing fat oxidation and decreasing carbohydrate consumption is important for enhancing endurance capacity during exercise.

In addition, several nutritional supplements such as caffeine, green tea extract, and L‐carnitine have been reported to improve endurance capacity by enhancing fatty acid oxidation (Kim et al., 2016). Therefore, exercise training combined with nutritional supplementation is an important strategy for improving endurance capacity because their effects on the metabolic adaptation are often additive.

*D*‐Allulose, also known as *D*‐psicose, is a rare sugar that is present in limited quantities in nature. *D*‐Allulose is a C‐3 epimer of *D*‐fructose, has approximately 70% of the sweetness of sucrose with almost zero calories, and has anti‐obesity and anti‐diabetic effects in humans and rodents (Han et al., 2016; Hossain, Yamaguchi, Hirose, et al., 2015; Itoh et al., 2015; Shintani et al., 2017). The potential mechanisms of the glucose‐lowering effect of *D*‐allulose involve decreased absorption of glucose via inhibition of α‐glucosidase (Hossain, Yamaguchi, Matsuo, et al., 2015) and upregulation of glycogen synthesis via glucokinase activation (Hossain, Yamaguchi, Matsuo, et al., 2015; Shintani et al., 2017). In addition, *D*‐allulose downregulates hepatic lipogenesis and enhances systemic energy expenditure and fatty acid oxidation (Chen et al., 2019; Kanasaki et al., 2021; Nagata et al., 2018). However, it is unknown how *D*‐allulose influences endurance capacity in exercise training. In this study, we aimed to examine the combined effect of exercise training and *D*‐allulose on endurance capacity in mice.

## 2 MATERIALS & METHODS

### 2.1 Animals and experimental design

All procedures were approved by the Meijo University Animal Care and Use Committee and conducted according to the guiding principle for the Care and Use of Laboratory Animals set forth by the Physiological Society of Japan (2019PE38). Male C57BL/6J mice were obtained from Japan SLC (Shizuoka, Japan). The mice were housed on a 12: 12 h light‐dark cycle in an environmentally controlled room (23 ± 1°C, 55 ± 5% relative humidity) and received food and water *ad libitum*. After 1 week of acclimation, the mice were fed either an AIN‐93G diet containing 3% (w/w) cellulose (Con) or 3% *D*‐allulose (Allu), provided by Matsutani Chemical Co., Ltd. (Itami, Japan) (Table 1). To equalize the calories of these diets, cellulose was used as the contrast condition for *D*‐allulose as it has almost zero calories. Additionally, the mice were assigned to the sedentary (Sed) or exercise training (Ex) groups (Con‐Sed, Con‐Ex, Allu‐Sed, Allu‐Ex, respectively; *n* = 6–7/group). Mice were single‐housed to enable accurate food intake measurements and prevent variability related to fighting and huddling.

**TABLE 1:** Composition of the experimental diets

Ingredients	Control diet (%)	*D*‐allulose diet (%)
Cornstarch	38.6	38.6
Casein	19.4	19.4
pregelatinized corn starch	12.8	12.8
Granulated sugar	9.7	9.7
Soybean oil	6.8	6.8
Cellulose	7.9	4.9
D‐allulose	0.0	3.0
Mineral mixture	3.4	3.4
Vitamin mixture	1.0	1.0
L‐cystine	0.3	0.3
Choline bitartrate	0.2	0.2
t‐Butylhydroquinone	0.001	0.001
Total	100	100

### 2.2 Exercise training and exhaustive running test

Mice in the two Ex groups (regardless of diet) were trained using a motor‐driven treadmill (MK‐680, Muromachi Kikai, Tokyo, Japan) 5 days per week for 4 weeks in a climate‐controlled room. The exercise was conducted during the dark (active) period. The 1st day of training began at a workout intensity of 10–12 m/min without grade for 30 min. The intensity and duration increased gradually until 18 m/min was attained for 60 min, considering the effects of exercise training. Electric shock was rarely used to motivate animals to run.

After the exercise training period, the mice in all groups underwent an exhaustive running test to assess their endurance capacity. Specifically, they were subjected to treadmill running at 12 and 15 m/min for 5 min each, followed by 18 m/min for 20 min, while the running speed was incrementally increased by 2 m/min every 20 min until exhaustion. Exhaustion was defined as the inability to continue regular treadmill running despite repeated tapping on the mouse's tail as stimulation. The running time was recorded as the endurance capacity. At 48 h after the exhaustive running test, the mice in the two Ex groups were subjected to run at 18 m/min for 60 min again and, then, anesthetized with isoflurane and sacrificed immediately after completing the exercise bout.

### 2.3 Histological analysis

Epididymal fat was excised and fixed overnight in 4% paraformaldehyde. Samples were paraffin‐embedded and 4‐μm thick sections were prepared and stained with hematoxylin and eosin (H&E). Section images were captured using a microscope (20×; BZ‐9000, Keyence, Osaka, Japan) and transferred to a computer. The area of 350–500 adipocytes was randomly measured using ImageJ software (NIH, Bethesda, MD, USA).

### 2.4 Measurement of glycogen in skeletal muscle and liver

Glycogen content in the gastrocnemius muscle and liver was measured using a commercially available glycogen assay kit (K646‐100, BioVision). Tissues were homogenized with ice‐cold dH_2_O, boiled for 10 min at 95°C, and centrifuged at 12,000 × g for 15 min at 4°C. The supernatant was aliquoted in duplicate and supplemented to 50 μl with hydrolysis buffer. A duplicate sample was used as a free glucose control (no addition of hydrolysis enzyme) and was subtracted from the determined concentration to calculate the final glycogen concentration. The assay was performed according to the manufacturer's instructions, and the plates were read at 570 nm on a FilterMax F5 (Molecular Devices, CA, USA). The glycogen content of each sample was normalized to the protein concentration used in each sample.

### 2.5 Sample preparation and immunoblotting

The gastrocnemius muscle and liver were powdered under liquid nitrogen and were homogenized in ice‐cold RIPA buffer (25 mM Tris‐HCl, pH 7.6, 150 mM NaCl, 1% NP‐40, 1% sodium deoxycholate, 0.1% sodium dodecyl sulfate; Thermo Fischer Scientific, Waltham, MA, USA) containing 10 mM EDTA, Halt^TM^ Protease inhibitor cocktail EDTA‐free (Thermo Fischer Scientific), and PhosSTOP (Roche, Penzberg, Germany). Homogenates were centrifuged at 12,000 g for 15 min at 4°C. Protein concentrations in the supernatants were determined using a BCA protein assay kit (Thermo Fischer Scientific). Protein extracts (2.0 mg/ml) were solubilized in sample buffer (30% glycerol; 5% β‐mercaptoethanol; 2.3% sodium dodecyl sulfate [SDS]; 62.5 mM Tris‐HCl, pH 6.8; and 0.05% bromophenol blue) and incubated at 95°C for 5 min.

Proteins were loaded onto 10% SDS polyacrylamide gel electrophoresis (SDS‐PAGE) gels and run at 150 V for 50–60 min. Next, the proteins were transferred to polyvinylidene difluoride (PVDF) membranes at 100 V for 1 h. The membranes were blocked for 1 h at room temperature using a PVDF blocking reagent (Toyobo Co. Ltd., Osaka, Japan). After three washes with Tween‐Tris‐buffered saline (T‐TBS: 40 mM Tris‐HCl, 300 mM NaCl, and 0.1% Tween 20; pH 7.5), the membranes were incubated with the following primary antibodies: phosphorylated glycogen synthase kinase 3β (GSK3β; 1: 2000; #5558; Cell Signaling Technology, Danvers, MA, USA), GSK3β (1: 2000; #12456; Cell Signaling Technology), phosphorylated Thr172‐adenosine monophosphate‐activated protein kinase (AMPK; 1: 2,000; #2535; Cell Signaling Technology), AMPK (1: 2,000; #2532; Cell Signaling Technology), phosphorylated Ser79‐acetyl coenzyme A carboxylase (ACC; 1: 2,000; #3661; Cell Signaling Technology), and ACC (1: 2,000; #3662; Cell Signaling Technology) in dilution buffer overnight at 4°C. After several washes in T‐TBS, the membranes were incubated with anti‐rabbit horseradish peroxidase (HRP)‐conjugated secondary antibodies (1: 20,000; #7074; Cell Signaling Technology) in dilution buffer for 1 h at room temperature. After several washes, the bands were visualized using the Immobilon Western chemiluminescent HRP substrate (Millipore Corporation, Billerica, MA, USA), and the signals were recorded using the Fusion FX (Vilber, Marne‐la‐Vallee, France). Analyses were performed using Evolution Capture software (Vilber). Protein phosphorylation was calculated as the ratio of the phosphorylated to the total protein levels and expressed as arbitrary units.

### 2.6 Statistical analysis

Data are expressed as means ± standard error (SE). Statistical significance was determined using a two‐way analysis of variance (ANOVA), Student's *t*‐test, or log‐rank Mantel‐Cox test (for running population and time to exhaustion). When the ANOVA results reached significance, group differences were assessed using Bonferroni's post hoc test. Statistical significance was set at *p* < 0.05. Statistical analyses were performed using PRISM software (ver. 6.0; GraphPad, San Diego, CA, USA).

## 3 RESULTS

### 3.1 Body weight and food intake during experimental periods, and organ weights

**FIGURE 1:**
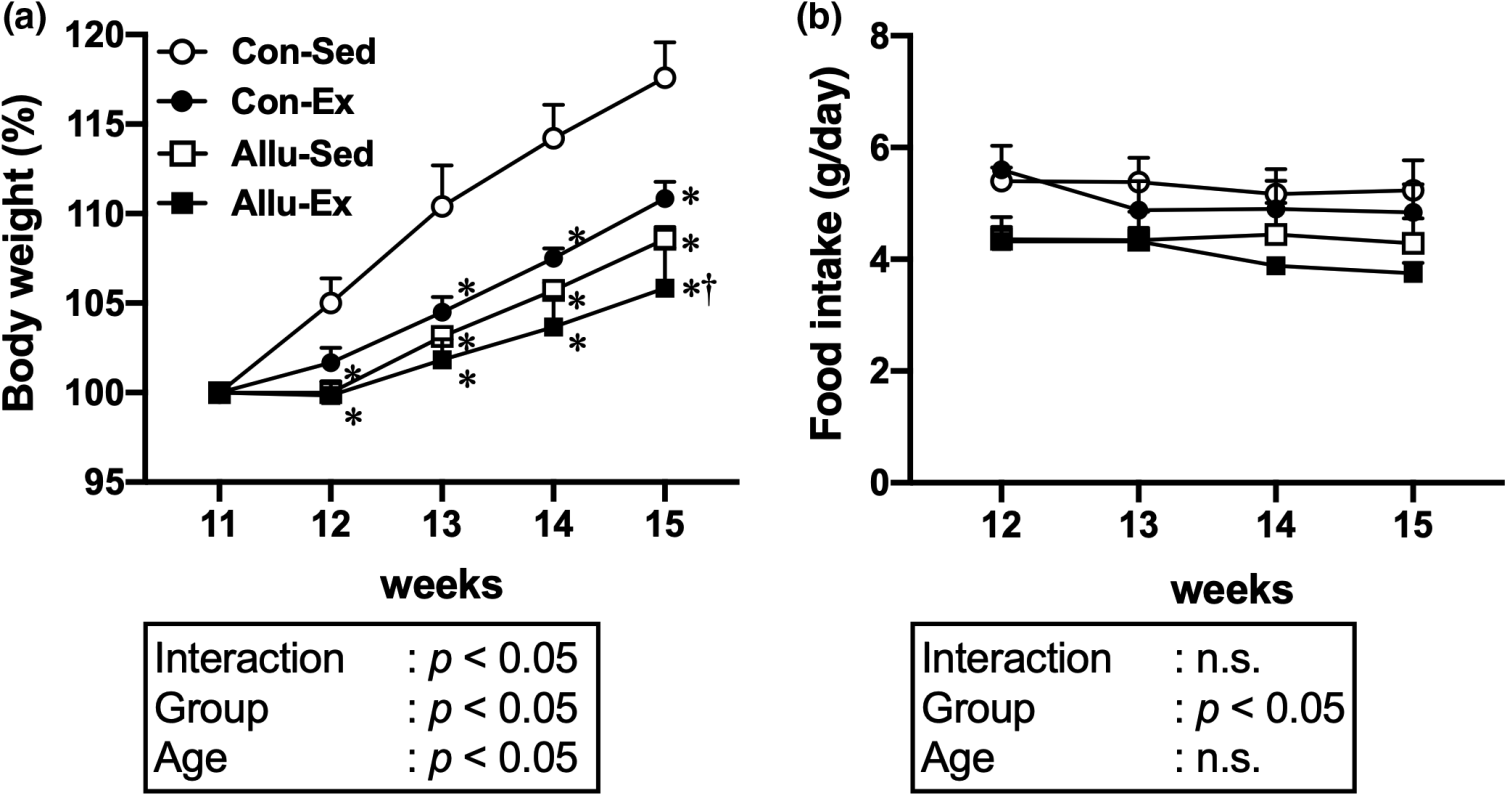
Changes in body weight and food intake during the experimental period. Body weight (a) and food intake (b). Values are shown as means ± standard error. ^*^ *p* < 0.05 versus Con‐Sed, ^†^ *p* < 0.05 versus Con‐Ex

Changes in body weight and food intake during the experimental period are shown in Figure 1. The relative weight gain was significantly less in the Con‐Ex, Allu‐Sed, and Allu‐Ex groups than in the Con‐Sed group (*p* < 0.05, Figure 1a). A significant main effect of the group was observed for food intake during the experimental period (*p* < 0.05, Figure 1b), with a tendency toward lower food intake in mice fed the *D*‐allulose diet than in those fed the control diet.

Table 2 shows the organ weights of the experimental groups. There were no significant differences between the groups in the weights of the liver and gastrocnemius muscles, whereas the weights of epididymal, perirenal, and subcutaneous fat were significantly lower in the Allu‐Ex than in the Con‐Sed group (*p* < 0.05). Perirenal and subcutaneous fat weights were also significantly lower in the Allu‐Sed than in the Con‐Sed group (*p* < 0.05).

**TABLE 2:** Organ weights in the experimental groups

	Con		Allu	
	Sed	Ex	Sed	Ex
Liver (mg)	1146.4 ± 72.5	1072.9 ± 86.1	1156.3 ± 85.1	1129.2 ± 59.5
Gastrocnemius muscle (mg)	140.7 ± 5.4	141.5 ± 7.3	139.2 ± 4.7	139.7 ± 7.3
Epididymal fat (mg)	785.3 ± 199.2	633.4 ± 179.6	634.9 ± 89.7	509.6 ± 99.3*
Perirenal fat (mg)	395.5 ± 125.5	296.6 ± 97.2	250.9 ± 33.3*	217.5 ± 29.7*
Subcutaneous fat (mg)	600.8 ± 170.9	468.5 ± 76.5	433.7 ± 55.5*	381.9 ± 30.1*

Note

Values are shown as means ± standard error.

* *p* < 0.05 versus Con‐Sed group.

### 3.2 Frequency distribution of adipocytes size

**FIGURE 2:**
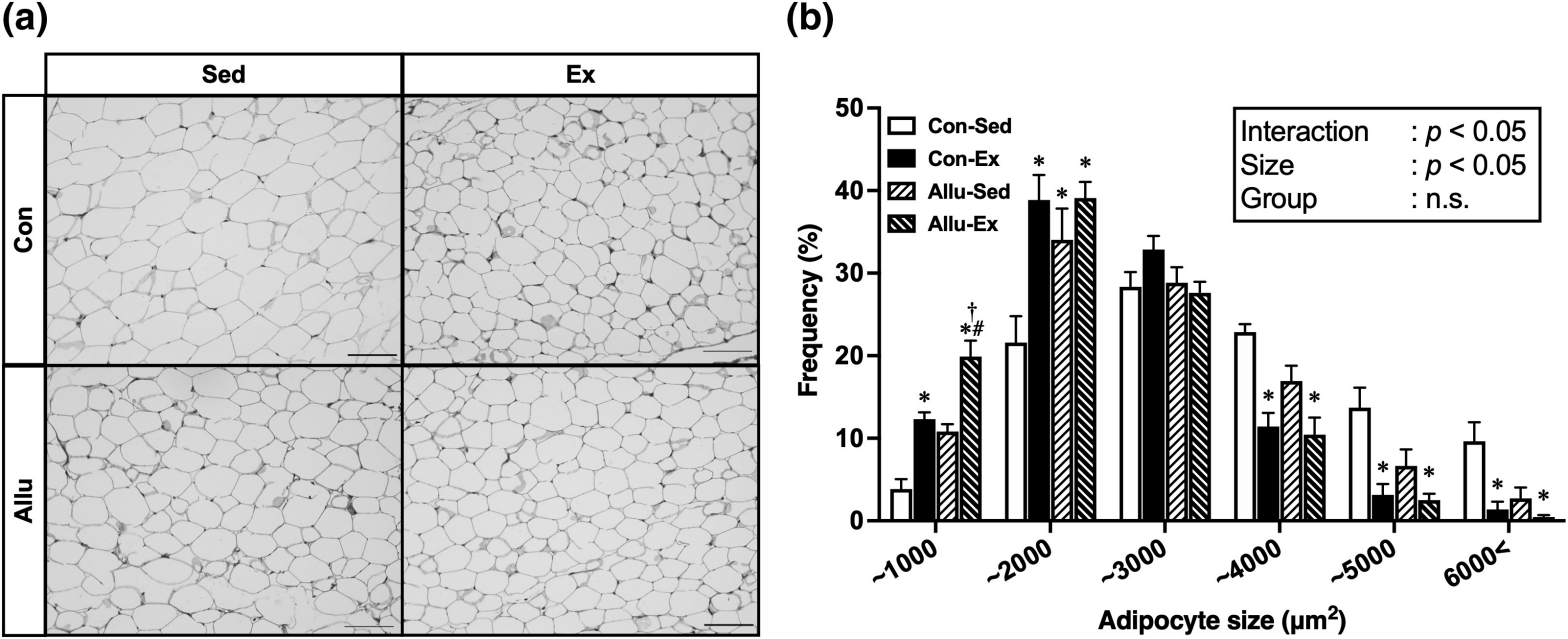
Effects of exercise training and *D*‐allulose intake on the adipocyte size in epididymal fat. H&E staining of epididymal fat sections (20× magnification, scale bar = 100 μm) (a). Histogram depicting the size distribution of the measured white adipocytes in epididymal fat (b). Values are shown as means ± standard error. ^*^ *p* < 0.05 versus Con‐Sed, ^†^ *p* < 0.05 versus Con‐Ex, ^#^ *p* < 0.05 versus All‐Sed. H&E, hematoxylin, and eosin

Next, we examined the adipocyte size in the epididymal fat by H&E staining (Figure 2). The frequency of smaller adipocytes (<1000 μm^2^) was significantly greater in the Allu‐Ex than in the other three groups (*p* < 0.05). Conversely, the frequency of larger adipocytes (>3000 μm^2^) was significantly lower in the Con‐Ex and Allu‐Ex groups than in the Con‐Sed group (*p* < 0.05).

### 3.3 Exercise capacity

**FIGURE 3:**
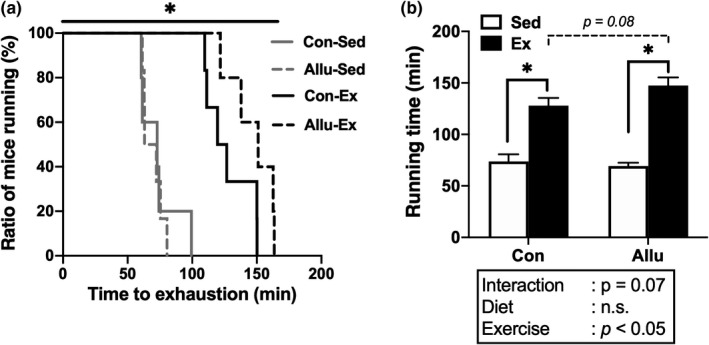
Effects of exercise training and *D*‐allulose intake on endurance capacity. Running population (a) and running time to exhaustion (b). Values are shown as means ± standard error. ^*^ *p* < 0.05

We measured the running time until exhaustion in all groups to assess endurance capacity. Exercise training markedly increased the running population of mice for a long time. The running population of mice was greater in the Allu‐Ex than in the Con‐Ex group (*p* < 0.05, Figure 3a). In addition, there were no significant differences in the running time to exhaustion between the diet conditions in the sedentary groups, whereas the running time tended to be longer in the Allu‐Ex than in the Con‐Ex group (*p* = 0.08, Figure 3b). Collectively, these results suggested that the *D*‐allulose diet upregulated endurance capacity induced by exercise training.

### 3.4 Glycogen content and GSK3β phosphorylation

**FIGURE 4:**
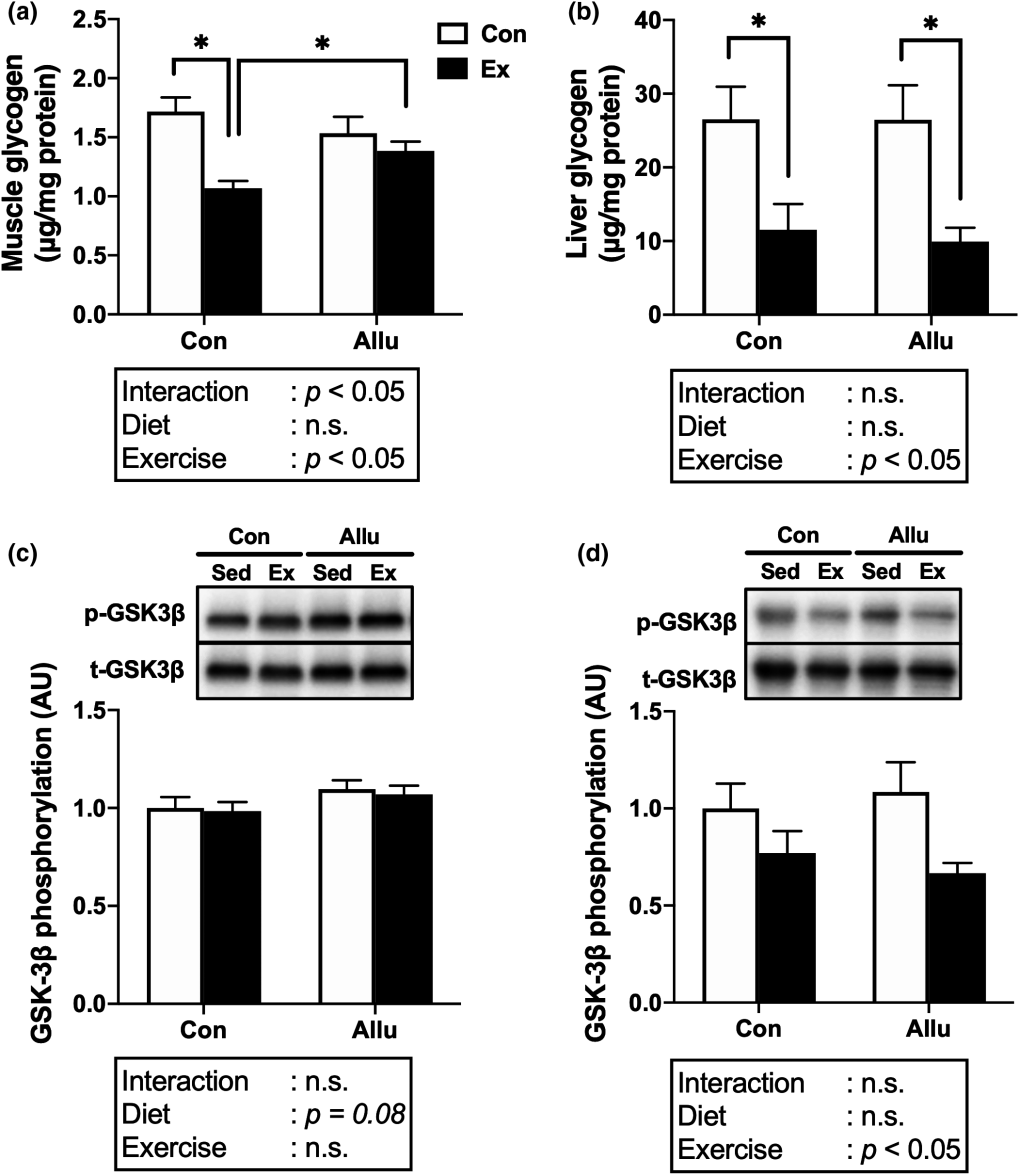
Effects of exercise training and *D*‐allulose intake on glycogen content and GSK3β phosphorylation. Glycogen content in the gastrocnemius muscle (a) and liver (b), and GSK3β phosphorylation in the muscle (c) and liver (d) after the final exercise bout Values are shown as means ± standard error. ^*^ *p* < 0.05. GSK, glycogen synthase kinase

We measured the glycogen content in the gastrocnemius muscle and liver. There were no significant differences in the glycogen content in the muscle and liver after receiving different diets in the sedentary groups. The muscle glycogen content was significantly lower in the Con‐Ex than in the Con‐Sed group (*p* < 0.05, Figure 4a) and was significantly higher in the Allu‐Ex than in the Con‐Ex group (*p* < 0.05), suggesting partial suppression of exercise‐induced glycogen consumption in the Allu‐Ex group. In contrast, the glycogen content in the liver was significantly lower in the two Ex groups than in the sedentary groups after receiving each diet (*p* < 0.05, Figure 4b). Moreover, there was no significant difference in the glycogen content in the liver between the Con‐Ex and Allu‐Ex groups.

Next, we analyzed the phosphorylation of GSK3β in the muscle and liver, a key regulator of glycogen synthesis. The GSK3β phosphorylation in the muscle tended to increase after receiving the *D*‐allulose diet but the exercise effect was not observed (Figure 4c). Conversely, there were no significant differences in the GSK3β phosphorylation in the liver between the different diet conditions, but a significant main effect of exercise was observed for the GSK‐3β phosphorylation, suggesting that the exercise decreased the GSK‐3β phosphorylation in the liver (Figure 4d).

### 3.5 AMPK‐ACC signaling

**FIGURE 5:**
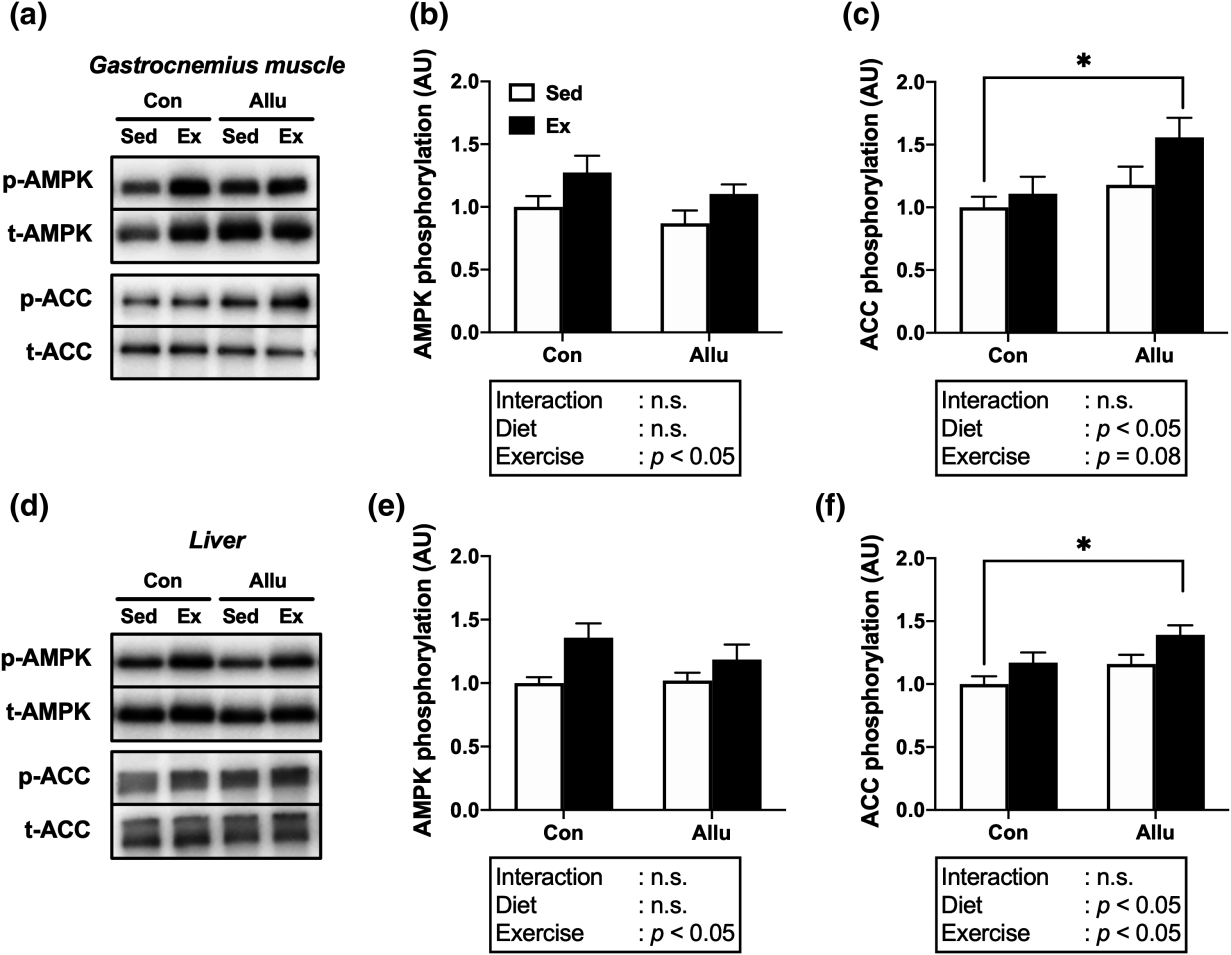
Effects of exercise training and *D*‐allulose intake on phosphorylation of the AMPK‐ACC signaling cascade. Representative western blots (a, d) and phosphorylation ratios of AMPK (b, e) and ACC (c, f) in the gastrocnemius muscle and liver after the final exercise bout. Values are shown as means ± standard error. ^*^ *p* < 0.05. AMPK, adenosine monophosphate‐activated protein kinase; ACC, acetyl‐coenzyme A carboxylase

In the gastrocnemius muscle, a significant main effect of exercise was observed for AMPK phosphorylation, suggesting that exercise can increase AMPK phosphorylation (Figure 5a,b). However, there was no significant main effect of *D*‐allulose diet. In contrast, a significant main effect of *D*‐allulose diet was observed for ACC phosphorylation, suggesting that *D*‐allulose can enhance ACC phosphorylation. In addition, exercise tended to increase ACC phosphorylation. The post‐hoc test revealed significantly higher ACC phosphorylation levels in the Allu‐Ex than in the Con‐Sed group (*p* < 0.05, Figure 5a,c).

Similar to that in the gastrocnemius muscle, a significant main effect of exercise was observed for AMPK phosphorylation in the liver (Figure 5d,e). Moreover, significant main effects of exercise and *D*‐allulose diet were observed for ACC phosphorylation in the liver. The post‐hoc test revealed significantly higher ACC phosphorylation levels in the liver in the Allu‐Ex than in the Con‐Sed group (*p* < 0.05, Figure 5d,f).

## 4 DISCUSSION

In this study, we investigated the combined effects of exercise training and *D*‐allulose intake on endurance capacity in mice as well as the underlying mechanisms. The main finding of the present study was that exercise training with *D*‐allulose intake enhanced endurance capacity, which might be attributed to the sparing of glycogen consumption in the skeletal muscle during exercise. In addition, the combination of exercise training and *D*‐allulose intake upregulated AMPK‐ACC signaling, which is associated with fatty acid oxidation, in the skeletal muscle and liver. The present study is the first to report the combined effects of exercise training and *D*‐allulose on exercise ability.

Several studies have reported that *D*‐allulose has anti‐obesity effects (Itoh et al., 2015; Ochiai et al., 2014). In the present study, the combination of exercise training and *D*‐allulose resulted in effective weight loss and reduced the adipocyte size. A previous study reported that energy expenditure and fatty acid oxidation, measured by indirectly using calorimetry, increased after 4 weeks of *D*‐allulose feeding (Nagata et al., 2015). Although the resting metabolic rates were not measured in this study, we speculate that the body weight and adipocyte size were reduced effectively as a result of increased energy expenditure at rest by *D*‐allulose intake and during exercise as well.

In the present study, exercise‐trained mice fed the *D*‐allulose diet attained higher endurance capacity. We assume that this was associated with the higher glycogen content in the muscle immediately after constant endurance exercise in the mice fed the *D*‐allulose diet. As muscle glycogen depletion leads to exercise‐induced exhaustion (Karlsson & Saltin, 1971), the sparing of glycogen consumption in the muscle during exercise might have contributed to enhanced endurance capacity. In contrast, previous studies have reported that liver glycogen plays a key role in the endurance capacity of mice (Gonzalez et al., 2016; Lopez‐Soldado et al., 2021), as genetical depletion of muscle glycogen did not lead to downregulate endurance capacity in mice (Pederson et al., 2005). However, in this study, there was no difference between the groups concerning the glycogen content in the liver after performing the final exercise bout. It is well known that GSK3β negatively regulates glycogen synthesis by phosphorylation of glycogen synthase. GSK3β itself is phosphorylated by an upstream kinase, such as Akt, and is inactivated, thus, resulting in increased glycogen synthesis (Rayasam et al., 2009). In this study, the GSK3β phosphorylation in the muscle slightly increased by *D*‐allulose diets. It may have been partially associated with sparing glycogen consumption in the muscle. Conversely, the GSK3β phosphorylation in the liver decreased after performing the final exercise bout. It is possible that suppression of glycogen synthesis in the liver and supply of glucose to circulation occurred because of maintaining the blood glucose levels during exercise.

In addition, the metabolic factors that affect endurance capacity comprise increased fat oxidation, which leads to the sparing of glycogen consumption in the muscle during exercise (Rennie et al., 1976). In the present study, glycogen content in the muscle was greater in trained mice with *D*‐allulose intake than in those without *D*‐allulose intake. *D*‐allulose may affect fat oxidation, resulting in the sparing of glycogen consumption and exercise‐induced enhancement of endurance capacity. The AMPK‐ACC signaling pathway is an important pathway regulating fatty acid oxidation and synthesis (Hardie & Pan, 2002). Exercise decreases the ATP levels and consequently increases the AMP/ATP ratio, leading to the activation of AMPK (Hardie et al., 2012). AMPK phosphorylates ACC, which catalyzes the irreversible carboxylation of acetyl‐CoA to produce malonyl‐CoA (Hardie & Carling, 1997; Park et al., 2002). Malonyl‐CoA‐generated ACC then inhibits carnitine palmitoyltransferase 1. Thus, ACC plays a crucial role in the rate‐limiting step of fatty acid synthesis and transport into the mitochondria. ACC phosphorylation reduces its enzymatic activity, thus leading to reduced fatty acid synthesis and enhanced fatty acid oxidation. In the present study, exercise increased AMPK phosphorylation in the skeletal muscle and liver, while exercise along with *D*‐allulose intake enhanced ACC phosphorylation in both tissues. ACC phosphorylation is better maintained and less transient than AMPK phosphorylation (Kraft et al., 2019). ACC phosphorylation is considered an appropriate readout of the activation of this pathway. Thus, our data showed that the combination of exercise and *D*‐allulose activates the AMPK‐ACC signaling pathway associated with lipid metabolism in the skeletal muscle and liver.

Our study had two major limitations. First, it remains unclear whether the combination of exercise training and *D*‐allulose diet can increase glycogen stores itself in the muscle and liver, as we did not measure the glycogen content after the training period without performing the final exercise bout. Second, we did not measure any direct markers of fatty acid oxidation. Thus, it is uncertain whether the combination of exercise training and *D*‐allulose diet actually enhanced fatty acid oxidation and contributed to the upregulation of endurance capacity. In consideration of these limitations, a further study should be conducted.

In summary, our data suggested that a combination of exercise training and *D*‐allulose intake is an effective strategy for upregulating endurance capacity. This may be associated with sparing glycogen content and enhancing activation of AMPK‐ACC signaling in the skeletal muscle.

## CONFLICT OF INTEREST

This study was funded by Matsutani Chemistry Industry Co., Ltd. (Itami, Japan). T. Y. and T. I. are employees of Matsutani Chemistry Industry Co., Ltd.

## AUTHOR CONTRIBUTION

Takamasa Tsuzuki, Takako Yamada, Tetsuo Iida, Bingyang Liu, Teruhiko Koike, and Yukiyasu Toyoda conceived and designed the research; Takamasa Tsuzuki, Ryo Suzuki, Risa Kajun, and Yukiyasu Toyoda performed the experiments; Takamasa Tsuzuki and Ryo Suzuki analyzed the data; Takamasa Tsuzuki, Takako Yamada, Tetsuo Iida, Teruhiko Koike, and Yukiyasu Toyoda interpreted the experimental results; Takamasa Tsuzuki and Ryo Suzuki prepared the figures; Takamasa Tsuzuki drafted the manuscript; Takamasa Tsuzuki, Takako Yamada, Tetsuo Iida, Bingyang Liu, Teruhiko Koike, Yukiyasu Toyoda, Takayuki Negishi, and Kazunori Yukawa edited and revised the manuscript; Takamasa Tsuzuki, Ryo Suzuki, Risa Kajun, Takako Yamada, Tetsuo Iida, Bingyang Liu, Teruhiko Koike, Yukiyasu Toyoda, Takayuki Negishi, and Kazunori Yukawa approved the final version of the manuscript.
